# Reports of Lunatic Hospitals

**Published:** 1849-05

**Authors:** 


### REPORTS OF LUNATIC HOSPITALS.

The annual report of two of these institutions is before us—that of the Pennsylvania Hospital for the Insane, by Dr. Kirkbride, and that of the Eastern Asylum of Virginia, by Dr. Galt. Both reports speak of a crowded state of the wards, and point out improvements demanded by the unfortunate class of patients consigned to them. Dr. Galt shows that the inmates of the Virginia hospital are, for the most part, victims of a hopeless insanity—confirmed lunatics, in whose cases medical treatment avails but little. These are, indeed, rendered far more comfortable in the hospital than would be their lot elsewhere, but if cures are to be made, he informs the legislature that they must be from among a different class of subjects. Of those under his care, only two have been insane less than a year, and only 19 from one to five years, while most have labored under the disease five, ten, twenty, or thirty years. The report of Dr. Galt is an intelligent document, and evinces in its author a praiseworthy zeal in the benevolent cause to which he is devoted.

The Pennsylvania hospital discharged, during the year 1848, two hundred and three patients, of whom 120 were cured, 23 were much improved, 24 improved, 19 were stationary, and 17 died. Of the patients discharged "cured" 57 had been only three months in the hospital; 35 between three and six months; 24 between six months and a year; and but four for a longer period than one year. Nothing could more forcibly illustrate the importance of subjecting the insane at the earliest period to the salutary regimen of a hospital. The statistics of Dr. Kirkbride's report are ample and instructive.

